# Female sex hormone, progesterone, ameliorates the severity of SARS-CoV-2-caused pneumonia in the Syrian hamster model

**DOI:** 10.1038/s41392-021-00860-5

**Published:** 2022-02-14

**Authors:** Lunzhi Yuan, Huachen Zhu, Kun Wu, Ming Zhou, Jian Ma, Rirong Chen, Qiyi Tang, Tong Cheng, Yi Guan, Ningshao Xia

**Affiliations:** 1 State Key Laboratory of Molecular Vaccinology and Molecular Diagnostics, National Institute of Diagnostics and Vaccine Development in Infectious Diseases, School of Life Sciences, School of Public Health, Xiamen University, Xiamen, Fujian China; 2 State Key Laboratory of Emerging Infectious Diseases, School of Public Health, Li Ka Shing Faculty of Medicine, The University of Hong Kong, Hong Kong, SAR China; 3 Department of Microbiology, Howard University College of Medicine, Washington, DC USA

**Keywords:** Infectious diseases, Infection

**Dear Editor**,

Severe acute respiratory syndrome coronavirus 2 (SARS-CoV-2) has infected more than 260 million people worldwide and causes coronavirus disease 2019 (COVID-19) with clinical spectrum ranging from mild to severe pneumonia. Recent clinical trials and experimental animal studies demonstrated that the severity of COVID-19 is lower in the females than in males.^1^ However, it is unclear whether sex hormones are associated with disease severity in COVID-19 patients. Previously, the anti-inflammatory effects of progesterone, a major female sex hormone, were observed in patients who suffered from the influenza virus-infected diseases.^2,3^ In this study, we aimed to investigate the role of progesterone in the Syrian hamster model of SARS-CoV-2 infection-caused lung pathogenesis. In order to model the male COVID-19 patients with severe pneumonia, male hamsters were intranasally infected with 1 × 10^4^ plaque-forming unit (PFU) of SARS-CoV-2 (Fig. 1a). The SARS-CoV-2-infected hamsters were untreated or treated with 1-, 3- or 5-dose of progesterone (1 mg/kg per dose) via intraperitoneal injection (Fig. 1a). All of the hamsters with or without progesterone therapy survived throughout the infection course. The SARS-CoV-2-infected hamsters without progesterone treatment exhibited progressive body weight loss of up to 12.6 ± 1.1% from 1 to 7 days post infection (dpi) (Fig. 1b). However, the SARS-CoV-2-infected hamsters treated with 1-, 3- or 5-dose of progesterone exhibited body weight loss of 9.6 ± 0.9%, 6.7 ± 1.1% or 5.1 ± 1.2% at 7 dpi, respectively (Fig. 1b). The uninfected hamsters with or without 5-dose of progesterone treatment showed a body weight increase from 0 to 7 dpi (Fig. 1b). These data suggested that progesterone rescued the body weight loss of the SARS-CoV-2-infected hamsters in a dose-dependent manner.

**Fig. 1:**
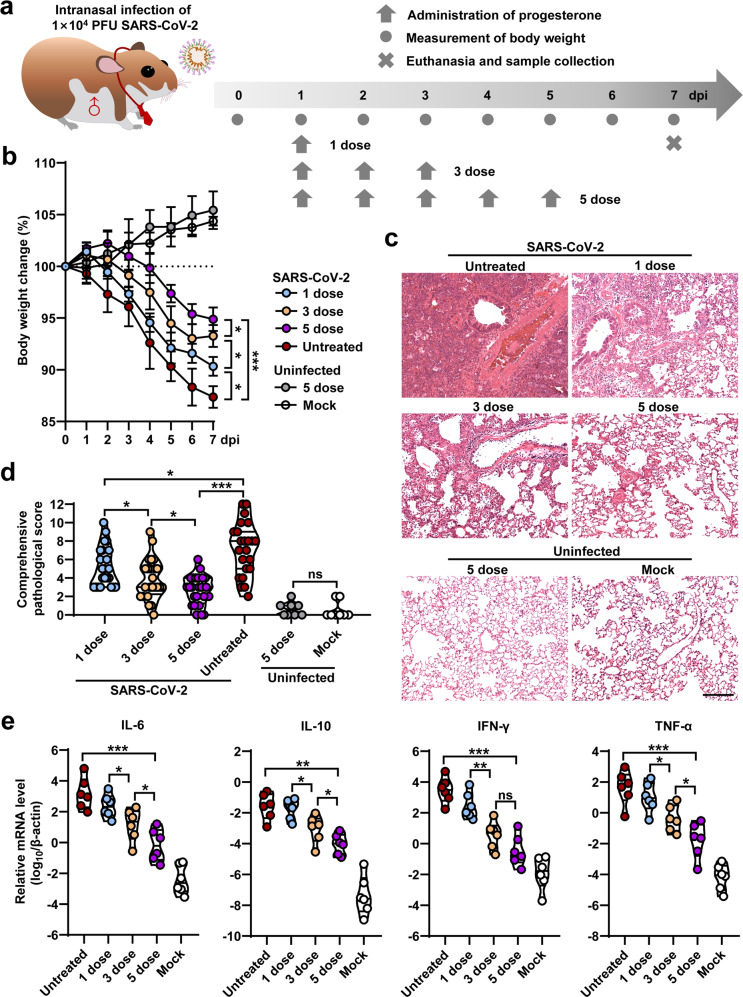
Detection of body weight, lung pathological changes and mRNA levels of proinflammatory cytokines in SARS-CoV-2 infected hamsters with or without progesterone treatment. **a** Schematic diagram of SARS-CoV-2 infection and animal operations. Hamsters were intranasally inoculated with 1 × 10^4^ PFU of SARS-CoV-2, then received intraperitoneal injection of 1-, 3- or 5-dose of progesterone, respectively. Body weight were daily observed. Animals were euthanized at 7 dpi for virological and histological analysis. The SARS-CoV-2 infected hamsters without progesterone treatment (Untreated) were set as positive control group. The hamsters without SARS-CoV-2 infection and the hamsters with 5-dose of progesterone treatment were set as negative control groups. **b** Body weight changes of hamsters from 0 to 7 dpi (*n* = 6). **c** Representative H&E staining for lung lobe sections collected from SARS-CoV-2 infected hamsters at 7 dpi (Bar = 200 μm). Comprehensive views of H&E staining were shown in Supplementary Fig. S1. **d** Comprehensive pathological scores for lung sections were determined based on the severity and percentage of injured areas for each lung lobe. **e** Fold changes for mRNA levels of proinflammatory cytokines include IL-6, IL-10, TNF-α, and IFN-γ in the lung tissues collected from SARS-CoV-2 infected hamsters with 5-dose progesterone treatment at 7 dpi (*n* = 6). The SARS-CoV-2 infected hamsters without treatment (Untreated) and the hamsters without SARS-CoV-2 infection (Mock) were set as control groups (*n* = 6). The mRNA levels of proinflammatory cytokines were standardized to the house-keeping gene γ-actin

To evaluate the severity of lung pathogenesis, viral load and host immune responses, all of the hamsters were euthanized at 7 dpi. Lung lobes were collected and fixed in formalin for systematic pathological analysis by hematoxylin and eosin (H&E) staining. The H&E staining results of lung lobes collected from SARS-CoV-2-infected hamsters that were untreated with progesterone showed typical features of severe pneumonia including increasing lung lobe consolidation and alveolar destruction, diffusive inflammation, protein-rich fluid exudate, hyaline membrane formation and severe pulmonary hemorrhage, which were not seen in the mock group (Fig. 1c and Supplementary Fig. 1). Interestingly, the H&E staining showed alleviated lung pathological changes for the progesterone treatment groups (Fig. 1c and Supplementary Fig. 1). The severity of lung pathogenesis is quantified by a comprehensive pathological score based on the observations of alveolar septum thickening and consolidation, hemorrhage, exudation, pulmonary edema and mucous, recruitment and infiltration of inflammatory cells among all of the hamster lung lobes. The mock or the SARS-CoV-2-infected hamsters without progesterone treatment showed average comprehensive pathological scores of 0.5 or 7.2 (Fig. 1d and Supplementary Table S1). The SARS-CoV-2-infected hamsters with 1-, 3- or 5-dose progesterone treatment showed average comprehensive pathological scores of 5.2, 3.9, or 2.7, respectively (Fig. 1d and Supplementary Table S1). Masson staining results of these sections suggesting progesterone therapy can suppress the SARS-CoV-2 induced lung fibrosis (Supplementary Fig. 2). Additionally, 5-dose progesterone treatment is able to alleviate SARS-CoV-2 induced body weight loss and lung pathogenesis in both male and female hamsters (Supplementary Fig. 3 and Supplementary Table S2). Whereas, 5-dose placebo showed no therapeutic effect (Supplementary Fig. 3 and Supplementary Table S2). In conclusion, the 5-dose progesterone treatment is adequate to restore SARS-CoV-2-induced lung injury in hamster model.

The major drivers of morbidity and mortality in COVID-19 are the dysregulated immune cells that excessively release proinflammatory cytokines such as interleukin 6 (IL-6), IL-10, tumor necrosis factor α (TNF-α), and interferon γ (IFN-γ).^4^ To know whether progesterone is able to suppress the these proinflammatory cytokines caused by SARS-CoV-2 infection, the mRNA levels of several proinflammatory cytokines from the homogenized lung tissues collected at 7 dpi were measured by RT real-time PCR (RT-qPCR). The SARS-CoV-2-infected hamsters showed ~2000- to 60,000-fold increase of mRNA levels of IL-6, IL-10, TNF-α, and IFN-γ as being compared to those form mock group (Fig. 1e). However, these stormily increased mRNA levels of proinflammatory cytokines were significantly suppressed by the 5-dose progesterone treatment (Fig. 1e). In addition, progesterone showed a dose-response for controlling proinflammatory cytokines (Fig. 1e). Then, we analyzed viral loads in respiratory tract organs including turbinate, trachea and lung by RT-qPCR that amplifies SARS-CoV-2 open reading frame 1ab (ORF1ab) and nucleocapsid gene (NP) for detection of viral RNA levels in the homogenized tissues collected at 7 dpi. The hamsters with 3- or 5-dose progesterone treatment showed a slight decrease of viral RNA load in lung tissues as being compared to the group of SARS-CoV-2-infected hamsters without treatment (Supplementary Fig. 4). No significant differences of viral RNA in turbinate and trachea were detected between the SARS-CoV-2-infected hamsters with and without progesterone treatment (Supplementary Fig. 4). Therefore, progesterone is able to suppress both the excessively released proinflammatory cytokines and viral replication in lung tissue of SARS-CoV-2-infected hamsters.

Besides regulation of fertility and menstruation, progesterone binds to its receptor that broadly expresses on the immune cells and suppresses the exuberant inflammatory responses.^5^ In this study, we suggested the potential use of progesterone for COVID-19 therapy in a hamster model of SARS-CoV-2 infection and lung pathogenesis. After SARS-CoV-2 infection, the sequential 5-dose progesterone rescued body weight loss, suppressed viral replication, and restored cytokine storm and lung injury. Moreover, progesterone can accelerate wound healing in respiratory epithelial cells by induction of amphiregulin.^2^ Combined with previous studies, our data imply that higher endogenous levels of progesterone may protect women from progressing to severe illness in COVID-19. Altogether, progesterone is an important biological factor that may modulate the gender bias of SARS-CoV-2 infection and pathogenesis, and might be able to serve as a potential therapeutic agent for COVID-19.

## Supplementary information

Supplementary Materials

## Data Availability

All data collected in this study are available from the corresponding authors upon reasonable request.
