# Supplementary material for: Female sex hormone, progesterone, ameliorates the severity of SARS-CoV-2-caused pneumonia in the Syrian hamster model
Source: Signal Transduct Target Ther. 2022 Feb 14;7:47. doi: 10.1038/s41392-021-00860-5 (PMC8844354; doi:10.1038/s41392-021-00860-5)
Supplement: Supplementary file 1 — Supplementary Materials [file 41392_2021_860_MOESM1_ESM.pdf]

Supplementary Materials

Female Sex Hormone, Progesterone, Ameliorates the Severity of SARS-CoV-2-Caused Pneumonia in the Syrian Hamster Model

Lunzhi Yuan[1#], Huachen Zhu[2#], Kun Wu[1#], Ming Zhou[1], Jian Ma[1], Rirong Chen[2], Qiyi Tang[3], Tong Cheng[1]*, Yi Guan[2]* and Ningshao Xia[1]*

Correspondence to:

Tong Cheng (tcheng@xmu.edu.cn)

Yi Guan (yguan@hku.hk)

Ningshao Xia (nsxia@xmu.edu.cn)

This PDF file include:

Materials and Methods

Experimental Animal and Biosafety

Preparation of Virus Stock

Virus inoculation and sample collection

Progesterone treatment

Detection of Viral RNA

Detection of cytokine mRNA

Histopathological Studies

Statistical Analysis

Supplementary Figure 1. Comprehensive views for H&E staining of lung lobes collected from the hamsters sacrificed at 7 dpi.

Supplementary Figure 2. Comprehensive views for Masson staining of lung lobes collected from the representative hamsters sacrificed at 7 dpi.

Supplementary Figure 3. Progesterone and placebo treatment in male and female hamsters infected with SARS-CoV-2.

Supplementary Fig. 4. Detection of viral RNA levels in respiratory tract organs.

Supplementary Table S1. Comprehensive lung pathological score of the control hamsters and the male hamsters with SARS-CoV-2 infection and progesterone treatment.

Supplementary Table S2. Comprehensive lung pathological score of the male and female hamsters with progesterone and placebo treatment.

Supplementary Table S3. The gene-specific primers (5' to 3') used for RT-PCR for cytokines.

Materials and Methods

Experimental Animal and Biosafety

The Golden Syrian Hamster was raised in the specific pathogen free animal feeding facilities. All the animal experiments were approved by the Medical Ethics Committee (SUCM2021-113). All experiments with infectious SARS-CoV-2 were performed in the biosafety level 3 (BSL-3) and animal biosafety level 3 (ABSL-3) facilities affiliated to the State Key Laboratory of Emerging Infectious Diseases, School of Public Health, The University of Hong Kong. Our staff wear powered air-purifying respirators that filter the air, and disposable coveralls when they culture the virus and handle animals that are in isolators. The researchers are disinfected before they leave the room and then shower on exiting the facility. All facilities, procedures, training records, safety drills, and inventory records are subject to periodic inspections and ongoing oversight by the institutional biosafety officers who consult frequently with the facility managers.

Preparation of Virus Stock

The SARS-CoV-2 ancestral strain AP-8 (hCoV-19/China/AP8/2020; GISAID accession number: EPI_ISL_1655937) was passaged on Vero cells. Viral stocks were prepared in Vero cells (#CCL-81, ATCC) with DMEM containing 2% FBS, 5ug/mL TPCK-trypsin, Penicillin-Streptomycin and 30mmol/L $MgCl_2$ (#11995, #10270106, #T1426 and #15140-122; purchased from GIBCO, SIGMA-ALDRICH and Invitrogen). Viruses were harvested and stored in ultra-low temperature refrigerator. The titers were determined by means of plaque assay in Vero cells.

Virus inoculation and sample collection

The hamsters were anesthetized by isoflurane (#R510-22, RWD Life Science) and the nasally inoculated with 1×10^4 PFU dose of SARS-CoV-2 diluted in 200uL PBS (#10010031, GIBCO). Body weight of these hamsters were measured by electronic balance. Blood was collected for detection of serum neutralizing antibody. Hamsters were treated with isoflurane lightly, after that, capillary tube was used to collect blood from orbital vein. Hamsters were euthanized at indicated time point for detection of

viral load and analysis of pathogenesis in lung lobes.

Progesterone treatment

The dry powder of progesterone (#ST1605, Beyotime) was dissolved in ethanol at a concentration of 1 mg/mL, and then immediately used for intraperitoneal injection. For each dose, hamster received 1 mg/kg of progesterone therapy.

Detection of Viral RNA

Supernatant of the tissue homogenate was used for detection of viral RNA. For the lung and trachea, 0.1g tissue was collected. For the turbinate, 0.1g tissue was collected. Viral RNA was extracted by using a QIAamp Viral RNA Mini kit (#52906, Qiagen) according to the manufacturer's instructions. The RT-qPCR was conducted by using the SLAN-96S Real-Time System (Hongshi, Shanghai, China) with a SARS-CoV-2 RT-qPCR Kit from Wantai (Beijing, China). Relative Viral RNA of SARS-CoV-2 ORF1ab gene and NP gene were determined using primers pairs and probes shown in the kit instruction. Viral RNA copies were expressed on a $\log_{10}$ scale after normalized to the standard curve obtained by using ten-fold dilutions of a SARS-CoV-2 stock.

Detection of cytokine mRNA

The lung tissues were cleaved into small pieces and soaked in RNAlater (#AM7021, Invitrogen). Total RNAs in lysed lung tissues were extracted with RNeasy Mini kit (#74106, Qiagen) and reverse-transcribed to cDNA with Fast-King Strand cDNA Synthesis Kit (#FP313, TIANGEN, Beijing) Diluted cDNAs (1:10) were quantified using SYBR Green I-based real-time PCR using the LightCycler® 480 instrument (Roche) per manufacturer's instructions. Threshold cycle (Ct) of each gene was normalized to the internal reference gene (hamster γ-actin) and comparative Ct (2-ΔΔCt) method was utilized to calculate changes in chemokine and cytokine gene expression profile. The gene-specific primers (5' to 3') used for RT-qPCR were listed in Supplementary Table S3.

Histopathological Studies

For pathological analysis, lung tissues were fixed in formalin for more than 48 hours, dehydrated and then embedded in paraffin wax. The wax block of lung tissues was cut into 4μm sections for several pathological staining and analysis. H&E staining was employed for analysis of general lung pathogenic lesions include pulmonary edema, consolidation and inflammation. Masson staining was performed for analysis of lung fibrosis according to the operating manual of a commercial kit (#MST-8004, Maxim

Biotechnology, Fuzhou, China). The standards for pathological score of lung tissues in this study are derived from our previous study in hamster model. Comprehensive pathological score of lung sections were performed according to the degree of lung lesions include alveolar septum hyperplasia, consolidation and impairment of alveolar structure, fluid exudation, mucus suppository, thrombus, inflammation recruitment and infiltration of immune cells in each individual lung lobes. For each hamster, three or four lung lobes were employed for evaluation of comprehensive pathological score. In brief, H&E staining result of each lung lobe was analyzed for its severity of pathological change. The pathological score include: a) Alveolar septum thickening and consolidation; b) Hemorrhage, exudation, pulmonary edema and mucous; c) Recruitment and infiltration of inflammatory immune cells. For each issue, score related to the severity: 0 indicate no pathological change was observed, 1 indicate moderate pathological change, 2 indicate mild pathological change, 3 indicate severe pathological change and 4 indicate very severe pathological change. In conclusion, scores of such three issues were added as the comprehensive pathological score of a lung lobe, and the average comprehensive pathological score of the lobes indicate the severity of lung pathogenesis in an evaluated hamster. The images of whole lung lobes were screened by a high-throughput screening microscope system (EVOS M7000, Invitrogen of Thermo Fisher Scientific).

Statistical Analysis

Student's unpaired two-tailed t-test and one-way ANOVA were performed using GraphPad Prism 8.0 (GraphPad Software). Data are presented as the means ± SD. Two-sided p-values <0.05 were considered significant: *P <0.05, **P <0.01, ***P <0.001, NS indicates no significance.

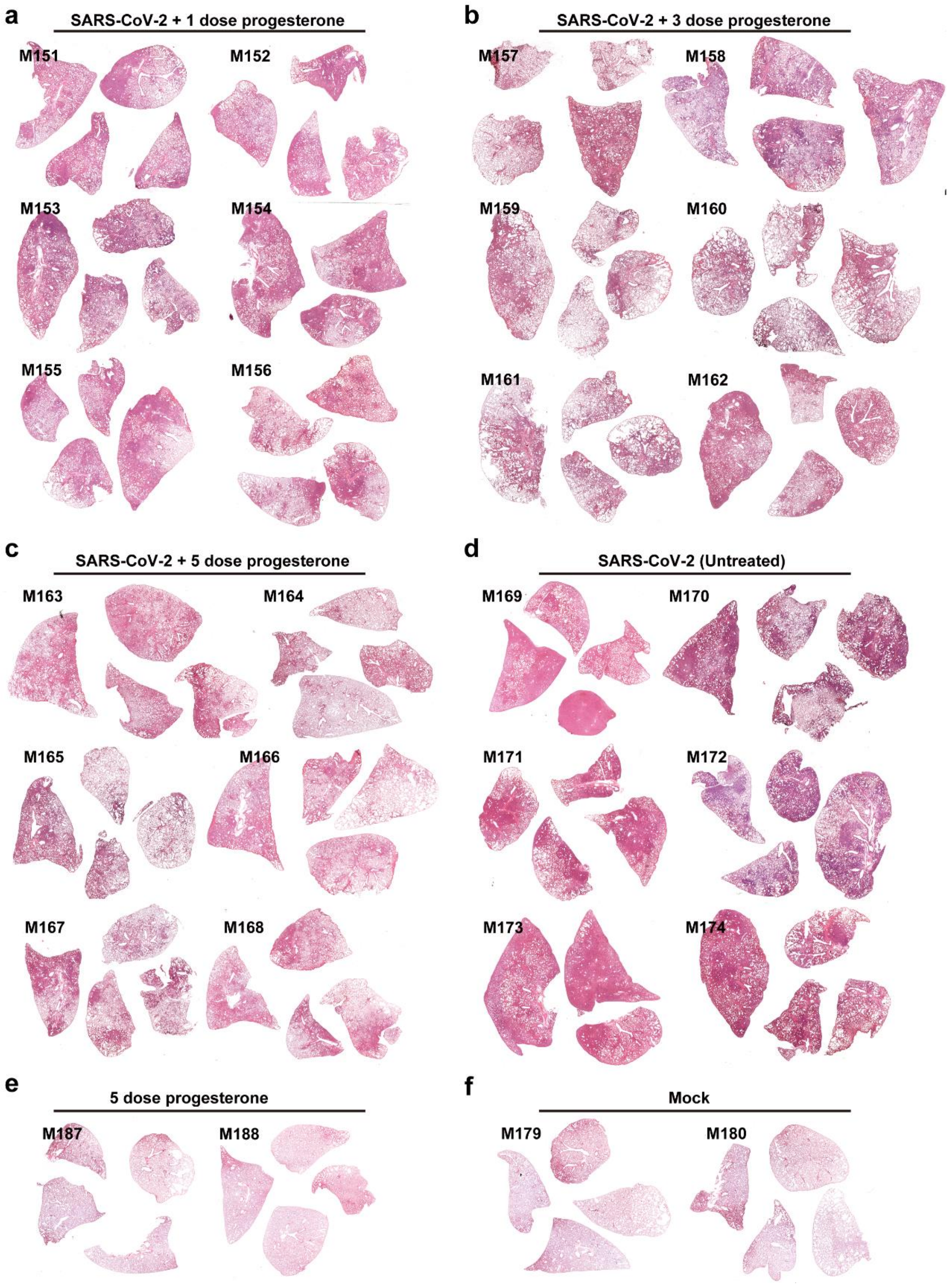

Supplementary Figure 1. Comprehensive views for H&E staining of lung lobes collected from the hamsters sacrificed at 7 dpi. All of the hamsters were sacrificed at 7 dpi. For each hamster, four lung lobes were fixed in formalin for pathological analysis. The hamster M154 and M173 showed only three lung lobes because of the operation errors during the manufacture procedure of paraffin sections. H&E staining for lung lobe sections collected from SARS-CoV-2 (AP-8/ancestral strain) infected

hamsters with **(a)** 1-, **(b)** 3- and **(c)** 5-dose progesterone treatment was screened by an auto-microscope system. **(d)** The lung lobe sections collected from SARS-CoV-2 infected hamsters without therapy were set as positive controls. The lung lobe sections collected from **(f)** hamsters without infection (mock) and **(e)** hamsters with 5-dose progesterone treatment were set as negative controls.

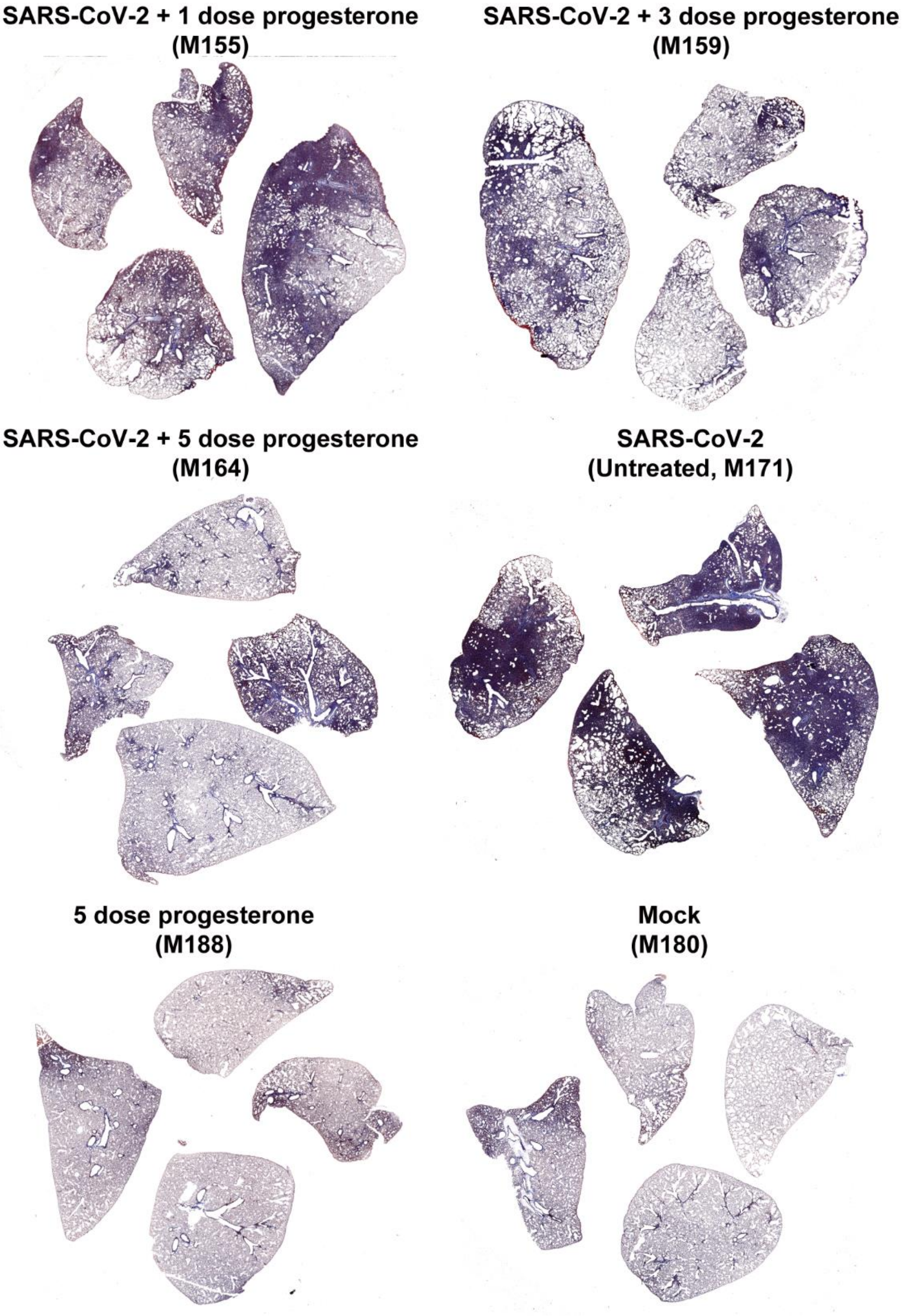

Supplementary Figure 2. Comprehensive views for Masson staining of lung lobes

collected from the representative hamsters sacrificed at 7 dpi. All of the hamsters were sacrificed at 7 dpi. For each hamster, four lung lobes were fixed in formalin for pathological analysis.

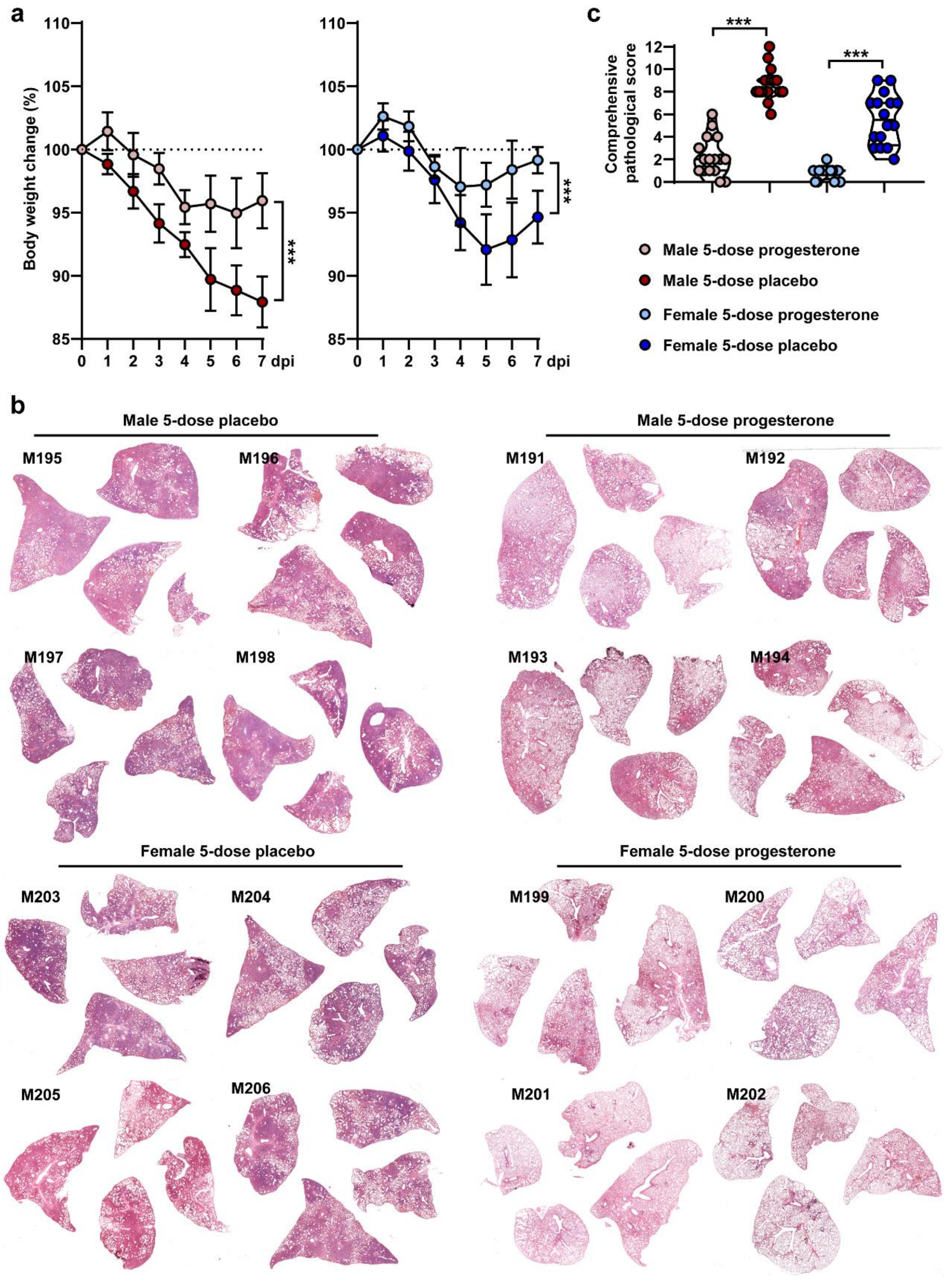

Supplementary Figure 3. Progesterone and placebo treatment in male and female

hamsters infected with SARS-CoV-2. Male and female were intranasally inoculated with 1×10^4 PFU of SARS-CoV-2, then received intraperitoneal injection of 5-dose progesterone or 5-dose placebo in equal volume (ethanol), respectively. Body weight changes of **(a)** male and female hamsters from 0 to 7 dpi (n=4). **(b)** Comprehensive views for H&E staining of lung lobes collected from the hamsters sacrificed at 7 dpi (n=4). **(c)** Comprehensive pathological scores for lung sections were determined based on the severity and percentage of injured areas for each lung lobe.

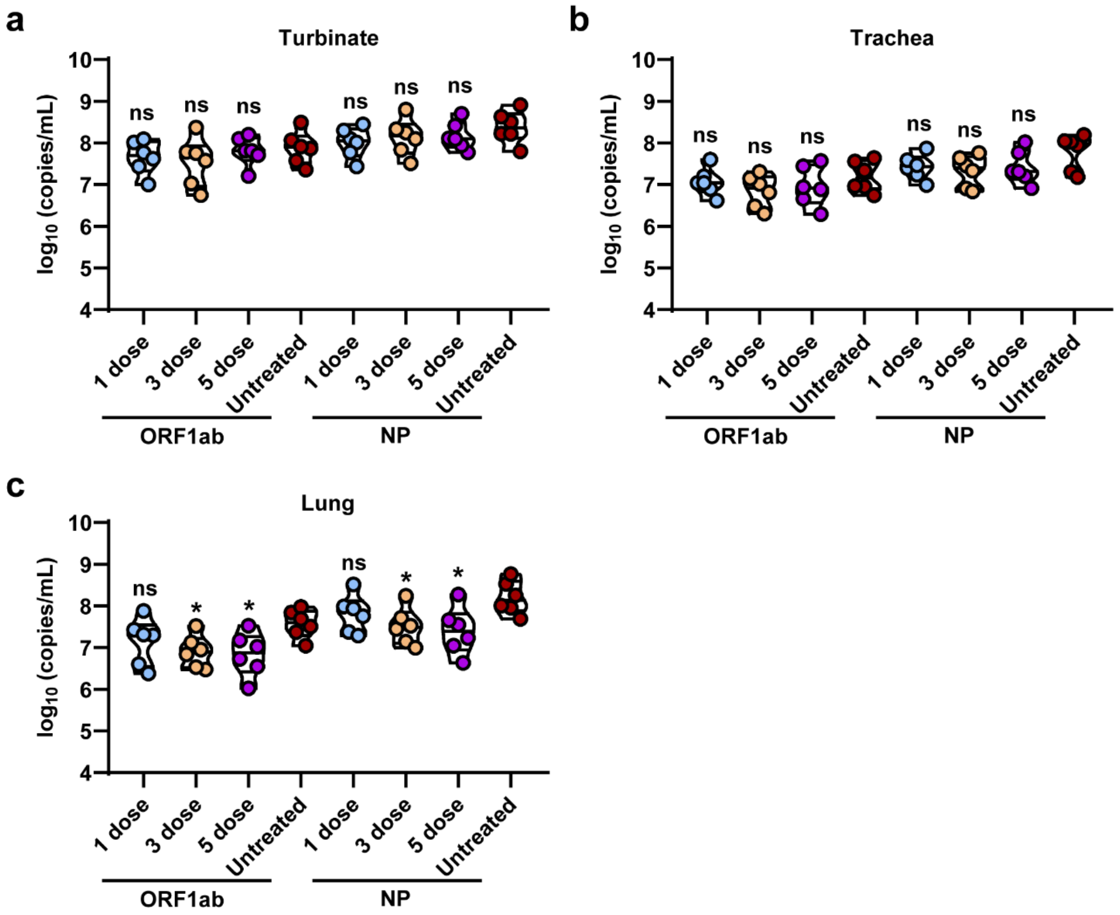

Supplementary Figure 4. Detection of viral RNA levels in respiratory tract organs. Viral RNA levels in **(a)** turbinate, **(b)** trachea and **(c)** lung collected from hamsters at 7 dpi were measured by RT-PCR (n=6). The primers of SARS-CoV-2 ORF1ab and NP genes were used.

Supplementary Table S1. Comprehensive lung pathological score of the control hamsters and the male hamsters with SARS-CoV-2 infection and progesterone treatment.

Group	Identifier	Pathological lesions			Comprehensive pathological score
		Alveolar septum hyperplasia and consolidation	Pulmonary edema, hemorrhage and mucus suppository	Recruitment and infiltration of inflammatory cells	
SARS-CoV2+1 dose progesterone	M151	2+1+1+1	2+2+1+1	2+1+1+1	6+4+3+3
	M152	3+1+1+1	3+2+2+1	4+2+1+1	10+5+4+3
	M153	2+2+1+1	3+2+2+1	3+3+2+1	8+7+5+3
	M154	3+2+2	3+2+2	3+3+2	9+7+6
	M155	2+1+1+1	3+2+2+1	3+2+1+1	8+5+4+3
	M156	2+1+1+1	2+2+1+1	3+1+1+1	7+4+3+3
SARS-CoV2+3 dose progesterone	M157	3+1+0+0	3+1+1+1	2+0+0+0	8+2+1+1
	M158	2+1+1+1	2+2+1+1	2+2+1+1	6+5+3+3
	M159	2+1+0+0	2+2+1+0	2+1+1+0	6+4+2+0
	M160	2+1+1+1	2+2+1+1	2+2+1+0	6+5+3+2
	M161	1+1+1+1	2+2+1+1	2+2+1+1	5+5+3+3
	M162	3+2+1+1	3+2+1+1	3+1+1+1	9+5+3+3
SARS-CoV2+5 dose progesterone	M163	1+1+1+1	2+2+1+1	1+1+1+0	4+4+3+2
	M164	1+1+0+0	1+1+0+0	1+0+0+0	3+2+0+0
	M165	1+1+0+0	2+2+1+0	1+1+0+0	4+4+1+0
	M166	2+1+1+0	2+1+1+1	1+1+0+0	5+3+2+1
	M167	2+1+1+0	2+1+1+1	2+1+1+0	6+3+3+1
	M168	1+1+1+1	2+2+1+1	1+1+1+0	4+4+3+2
	M169	4+3+1+1	4+3+2+2	4+4+1+1	12+10+4+4

**SARS-CoV2 (Untreated)**	**M170**	2+2+1+1	3+3+1+1	3+3+1+1	8+8+3+3
	**M171**	4+3+3+2	3+3+3+2	4+3+3+2	11+9+9+6
	**M172**	3+2+1+1	3+3+2+2	4+3+3+2	10+8+6+5
	**M173**	4+2+2	4+3+2	4+2+3	12+7+7
	**M174**	2+2+1+1	3+3+2+1	3+3+2+1	8+8+5+3
**5 dose progesterone**	**M187**	1+1+0+0	0+0+0+0	0+0+0+0	1+1+0+0
	**M188**	1+1+0+0	1+0+0+0	0+0+0+0	2+1+0+0
**Mock (Uninfected)**	**M179**	1+0+0+0	1+0+0+0	0+0+0+0	2+0+0+0
	**M180**	1+0+0+0	1+0+0+0	0+0+0+0	2+0+0+0

Supplementary Table S2. Comprehensive lung pathological score of the male and female hamsters with progesterone and placebo treatment.

**Group**	**Identifier**	**Pathological lesions**			**Comprehensive pathological score**
		**Alveolar septum hyperplasia and consolidation**	**Pulmonary edema, hemorrhage and mucus suppository**	**Recruitment and infiltration of inflammatory cells**	
**Male 5-dose progesterone**	**M191**	1+1+1+0	1+1+0+0	0+0+0+0	2+2+2+0
	**M192**	2+1+1+1	2+1+1+0	2+1+0+0	6+3+2+1
	**M193**	2+1+0+0	2+1+1+0	1+0+0+0	5+2+1+0
	**M194**	2+2+0+0	1+1+1+1	1+1+0+0	4+4+1+1
**Male 5-dose placebo**	**M195**	3+3+3	3+3+3	3+3+3	9+9+9
	**M196**	3+3+2+2	4+3+3+3	4+3+3+2	11+9+8+7
	**M197**	3+3+2+2	3+2+3+2	4+3+3+2	10+8+8+6
	**M198**	4+2+2+2	4+3+3+3	4+3+3+3	12+8+8+8
	**M199**	0+0+0+0	1+1+1+1	0+0+0+0	1+1+1+1

**Female 5-dose progesterone**	**M200**	0+0+0+0	1+1+1+1	0+0+0+0	1+1+1+1
	**M201**	0+0+0+0	0+1+1+0	0+0+0+0	0+1+1+0
	**M202**	1+0+0+0	1+1+1+0	0+0+0+0	2+1+1+0
**Female 5-dose placebo**	**M203**	3+3+1+1	3+3+2+1	3+3+2+1	9+9+5+3
	**M204**	2+2+1+1	2+2+1+1	3+2+1+1	7+6+3+3
	**M205**	3+3+2+1	2+2+2+1	2+2+1+0	7+7+5+2
	**M206**	2+2+1+1	3+2+1+1	3+3+2+2	8+7+4+4

Supplementary Table S3. The gene-specific primers (5' to 3') used for RT-PCR for cytokines.

**Genes**	**Forward**	**Reverse**
**Hamster IFN-γ**	TGTTGCTCTGCCTCACTCAGG	AAGACGAGGTCCCCTCCATTC
**Hamster IL-6**	AGACAAAGCCAGAGTCATT	TCGGTATGCTAAGGCACAG
**Hamster IL-10**	GGTTGCCAAACCTTATCAGAAATG	TTCACCTGTTCCACAGCCTTG
**Hamster TNF-α**	TGAGCCATCGTGCCAATG	AGCCCGTCTGCTGGTATCAC
**Hamster γ-actin**	ACAGAGAGAAGATGACGCAGATAATG	GCCTGA ATGGCCACGTACA
